# Disclosing genetic research results: experiences of the Colon Cancer Family Registry

**DOI:** 10.1186/1897-4287-9-S1-P18

**Published:** 2011-03-10

**Authors:** Louise Keogh, Douglass Fisher, Sheri Schully, Jan Lowery, Dennis Ahnen, Judi Maskiell, Noralane Lindor, John Hopper, Terrilea Burnett, Spring Holter, Sheri Sheinfeld Gorin, Pam Sinicrope, for the Colon Cancer Family Registry

**Affiliations:** 1 Centre for Women’s Health, Gender and Society, University of Melbourne, Melbourne, Victoria, Australia; 2 Fred Hutchinson Cancer Research Center, Seattle, Washington, USA; 3 Epidemiology and Genetics Research Program, Division of Cancer Control and Population Sciences, National Cancer Institute, Bethesda, Maryland, USA; 4 Department of Epidemiology, University of Colorado, USA; 5 Division of Gastroenterology, University of Colorado, USA; 6 Centre for Molecular, Environmental, Genetic and Analytic Epidemiology, University of Melbourne, Victoria, Australia; 7 Department of Medical Genetics, Mayo Clinic, Rochester, Minnesota, USA; 8 Public Health Sciences, Fred Hutchinson Cancer Research Center, Seattle, Washington, USA; 9 University of Hawaii, Manoa, Hawaii, USA; 10 Mount Sinai Hospital, Zane Cohen Centre for Digestive Diseases, Toronto, Ontario, Canada; 11 Department of Epidemiology, Joseph L. Mailman School of Public Health of Columbia University, New York, New York, USA; 12 Department of Psychiatry and Psychology, Behavioral Health Research Program, Cancer Center, Mayo Clinic, Rochester, Minnesota, USA

## Background

Literature on the ethics of returning research-generated genetic results to research participants has not reported on the practical experience of this activity. The Colon Cancer Family Registry (Colon CFR) has recruited participants from the US, Canada, Australia and New Zealand. Colon CFR-wide molecular testing has identified deleterious germline mutations in a DNA mismatch repair (MMR) gene for members of 424 families (153 *MLH1*, 206 *MSH2*, 39 *MSH6*, 26 *PMS2*). Carriers of mutations in these genes are at high risk of colorectal, endometrial and other cancers.

## Aim

To document our diverse experiences in delivering clinically important genetic results and the uptake of genetic results by participants.

## Methods

When a deleterious MMR gene mutation is identified in a family member, predictive testing is conducted on all enrolled relatives of the carrier, and a letter offering to disclose this information is sent to all family members. If participants choose to receive their results, genetic counseling is provided to participants free of charge. Protocols for the four sites currently offering to return genetic results are shown in Table 1.

**Table 1:** Protocols for returning genetic results

Steps in the protocol	Ontario	Mayo	Australasia	Hawaii
**When do sites inform participants that genetic results may be available**	At enrolment	At enrolment	At enrolment	When results available
**Who provides counseling (Genetic Counselor = GC)**	GC shared by study and hospital	MD or GC	government-funded GC service	GC employed by study
**Number of sessions**	2	2	2	2
**Mode of delivery of genetic results**	In person	Telephone & mail	In person	In person

## Results

Uptake of genetic test results by participants of families with MMR gene mutation results available ranged from 53-78%, (p=0.0001) (see Table 2).

**Table 2:** Uptake of genetic results

	Australasia (1999-2009)	Mayo (2008-2010)	Ontario (1998-2010)	Hawaii (1998-2010)
MMR mutation results available	805	185	260*	17*
Had genetic counselling	504	145	197	12
Received results	493	144	179	9
Decision pending	15	12	23	3
Uptake	61%	78%	69%	53%

* Probands only

## Discussion

The variation in uptake of genetic information could be related to the variation in the potential for insurance discrimination and/or the differences in the cost to consumers of genetic testing in the research and clinic setting.

## Conclusions

The return of genetic results and collection of uptake data has provided valuable information about the translation of these research findings and has led to translational research proposals. Delivering research-generated genetic results in the research setting, especially when sampling is population-based, provides both challenges and opportunities.

